# Understanding family caregivers’ needs to support relatives with advanced progressive disease at home: an ethnographic study in rural Portugal

**DOI:** 10.1186/s12904-020-00583-4

**Published:** 2020-05-25

**Authors:** Maria João Cardoso Teixeira, Wilson Abreu, Nilza Costa, Matthew Maddocks

**Affiliations:** 1 Royal National Orthopaedic Hospital NHS Foundation Trust & National Institute for Health Research (NIHR), Brockley Hill Road, Stanmore, Middlesex HA7 4LP UK; 2 School of Nursing & Research Centre “Centre for Health Technology and Services Research / ESEP -CINTESIS”, Porto, Portugal; 3 University of Aveiro - Research Centre “Didactic and Technology in the Education of Educators/CIDTFF”, Aveiro, Portugal; 4 Cicely Saunders Institute of Palliative Care, Policy & Rehabilitation, Florence Nightingale Faculty of Nursing, Midwifery & Palliative Care, King’s College of London, London, UK

**Keywords:** Advanced disease, Ethnography, Family caregiver, Home, Interviews, Needs, Nursing, Observations, Qualitative research

## Abstract

**Background:**

Family caregivers play an important role supporting their relatives with advanced progressive disease to live at home. There is limited research to understand family caregiver needs over time, particularly outside of high-income settings. The aim of this study was to explore family caregivers’ experiences of caring for a relative living with advanced progressive disease at home, and their perceptions of met and unmet care needs over time.

**Methods:**

An ethnographic study comprising observations and interviews. A purposive sample of 10 family caregivers and 10 relatives was recruited within a rural area in the north of Portugal. Data were collected between 2014 and 16 using serial participant observations (*n* = 33) and in-depth interviews (*n* = 11). Thematic content analysis was used to analyse the data.

**Results:**

Five overarching themes were yielded: (1) provision of care towards independence and prevention of complications; (2) perceived and (3) unknown caregiver needs; (4) caregivers’ physical and emotional impairments; and (5) balancing limited time. An imbalance towards any one of these aspects may lead to reduced capability and performance of the family caregiver, with increased risk of complications for their relative. However, with balance, family caregivers embraced their role over time.

**Conclusions:**

These findings enhance understanding around the needs of family caregivers, which are optimally met when professionals and family caregivers work together with a collaborative approach over time. Patients and their families should be seen as equal partners. Family-focused care would enhance nursing practice in this context and this research can inform nursing training and educational programs.

## Background

Globally, chronic diseases cause 38 million deaths each year, of which half occur in people aged over 70 [1]. In the European Union it has been estimated that the proportion of people aged over 65 would increase from 17% in 2005 to 30% in 2050 [2], with consistent patterns in Portugal [3]. In addition, in most of the European countries the percentage of home deaths has increased [4]. Care for people at home is often provided by family and friends [5]. Therefore, it is important to understand family caregivers’ roles when they act within the home environment [6].

In 2015, the World Health Organization argued that families must become progressively involved as care partners of their relative [7]. At the same time however, the ageing population means family caregivers will themselves increasingly become recipients of care [8]. Healthcare professionals face the challenge of supporting and improving families’ ability to care for people with chronic advanced disease [9]. Furthermore, professionals should help family caregivers to understand and assess their own needs as an important element of this process [10, 11]. Accordingly, family caregivers should be empowered to help reduce the risk of burnout [12].

Studies related to family caregivers identify a considerable level of need [13]. Home-based education can provide an opportunity for family caregivers to learn, develop specific knowledge and attitudes within a closed environment [14]. Successful education can support the development of family caregiver skills and positive attitudes towards caring for a relative in the home [15]. However, most of the time, home care is provided by community nurses without training in palliative care, particularly in rural areas away from tertiary centres [16].

Most studies in this field to date are from countries with high ranking palliative care metrics [17] and as such an evidence gap remains around the specific role and needs of family caregivers in rural community settings [18]. The need for a deep understanding of the Portuguese sociocultural context is necessary to achieve a high standard in caring and this awareness was the trigger for this study.

This paper reports on the first phase of a study entitled “Family Caregivers of People with Advance and Progressive Disease at Home: Contributions to a Model of Supervision”, developed to support community nurse in decision-making [19].

## Methods

### Aim

To explore family caregivers’ experiences when caring for a relative with advanced progressive disease at home, and to identify their perceived met and unmet needs, and understand care provided over time.

### Study design

An ethnographic study was conducted using qualitative data extracted from continuous observations and interviews with family caregivers on their experience of care. This methodology allows a contextual and reflexive approach to understand meanings, beliefs and feelings. The setting was operationalized where the social action occurred and it was essential to understand the perceptions, family caregivers’ approaches and performance. This method was deemed most appropriate due to its suitability to examine complex practices in real-life settings in which the researcher has little control, such as care provided at home [20].

### Participants and settings

The study was conducted in a rural area in the north of Portugal, where the palliative care and community services are scarce. Family caregivers of people with advanced progressive disease being cared for at home were recruited. Sampling was achieved through referrals from community doctors and nurses. A family caregiver was defined as “*a person responsible for the prevention and treatment of family members’ illness or disability”* [21]. Participants were considered eligible if they were adult, the person they cared for was over 65 years of age, with an incurable advanced disease and dependent in at least one dimension of self-care, and they provided most of their care, without any financial payment. Self-care was defined as “*Taking care of what is needed to maintain oneself, keep oneself going and handle basic individual and intimate necessities and activities in daily life”* [21].

Family caregivers under 18 years old were excluded as well as patients under 65 years old, with a curable disease and independent in self-care. The research team had no access to details on the excluded participants because the potential participants’ referrals were made by the community doctors and nurses, who were aware of the inclusion and exclusion criteria. None of the participants withdraw or declined to take part in the study.

### Data collection

#### Barthel scale

The Barthel scale was initially used to characterise dependence on the self-care of the person with advanced disease. This instrument evaluates: bowel and bladder control, personal hygiene, toilet use, feeding, transfer from bed to chair and vice versa, moving, dressing, going up and down stairs and bathing. Scoring is used to categorise the person as independent, dependent or totally dependent in self-care. This instrument was culturally validated and permission for use was obtained [22].

#### Participant observation

The Spradley methodology [20] was used for participant observations to provide an overview in their natural setting. Observations were taken in multiple spaces of the house, including the bedroom, bathroom, living room and kitchen. The researcher observed family caregivers when physical care was being provided. During these observations, a comprehensive interaction was maintained with the family caregivers, allowing the researcher to experience situations and events that were significant to them. Some observations were triggered by the family caregivers who called and asked the researcher to take part of family events. Throughout this phase, data were also obtained using informal conversations in order to understand the context of care, health status of care recipient and interaction with participants’, plus enquire about the expectations around the healthcare team, needs, and difficulties expressed during the caring process, and management of mood and emotions. An audio-record was used as a reflective diary to record the researcher’s observations. In the field notes, self-observations, self-doubts and reflections about conversations with the family caregivers and their relatives were recorded.

Additionally, the chronology of observations, tone and flow, plot and emotions has also been noticed. All observations took place between 2014 and 2016. Data collection continued until data saturation was reached [20, 23]. Observations were conducted over a period of 9 months in each home and covered weekends, evenings, and bank holidays. Across all study participants, over 120 h of observations took place.

#### In depth interviews

To gain insight from family caregivers, ethnographic semi-structured interviews were conducted using opened questions in our native language (Portuguese) and no translation was required, lasting 30–60 min each. Interviews were conducted by one researcher throughout in private. They aimed to create an open dialogue to clarify some points derived from observations, disperse any doubts, and explore in depth themes which were not covered during observations.

Some themes were explored through the interview in the absence of the relative such as: when they achieved awareness of the process of becoming a caregiver; motivations and reasons to take care; personal strategies to adjust and any new needs that had emerged over the course of the study, difficulties experienced, and any comments the caregiver wished to make. Interviews took place between during 2015. No paid caregivers or professionals involved in the care were presented during any of the observations or interviews.

### Data analysis

Immediately after each observation and interview, the researcher transcribed verbatim audio-recorded data. Participant observations and interviews were analysed according to the six phases of thematic analysis offered by Braun and Clarke [24] and checked against the records to ensure accuracy [24]. Transcripts were read thoroughly numerous times to ensure the researcher had become familiar with the content [25]. It was not possible to obtain participant validations of written transcriptions due to the low levels of literacy in the study population.

In relation to data from the observations, firstly, general interpretations and reflections were made [20]. The first coding was made using thematic analysis [26]. Subsequently, more focussed observations were conducted, related to the culturally sensitive subthemes. Subsequently, these subthemes were tabulated according to whether codes were obtained from observation, interviews or both. The coding process was applied to ensure validity and rigour and key themes were formed from a large number of examples within data [24]. After establishing key themes, codes under each key theme were reviewed and gathered into smaller themes. Interviews were also codified using thematic analysis [24]. Data analysis was supported by a qualitative analysis program – WebQDA [27].

### Rigour

Each transcription was initially analysed by the lead author and reviewed by the second and third authors to verify the themes generated from the data collected. Research trustworthiness was ensured using four criteria: credibility, transferability, dependability and confirmability [28]. To obtain participants’ most accurate responses, the researcher created a comfortable atmosphere to conduct observations and interviews. The researcher was a female doctoral student with 16 years of experience as a nurse and 5 years as a researcher. The study was reported in a comprehensive and transparent way by the aid of the COREQ checklist (supplementary file).

## Results

Ten family caregivers and 10 relatives living with advanced progressive disease participated in the study (Table 1). Family caregivers ranged from 46 to 78 years of age. Nine of the family caregivers were married female and were cohabiting. All were caring for their relatives 24 h per day and had done for periods ranging from 1 to 20 years. Five caregivers were siblings of the person living with advanced disease, two were daughter-in-law, and one each were sister, husband and niece. Nine were unemployed and providing care for their relative was the main reason caregivers aged under 65 years had not gained employment. All of them reported experiencing personal health changes over the years.

**Table 1:** Demographic Characteristic

	Person with Advanced Disease	Family Caregiver
**Age, years** Mean (range)	84 (72–93)	59 (46–78)
**Gender**		
Female / male	8 / 2	9 / 1
**Education**		
Illiterate	1	1
Elementary school	7	7
Illiterate	2	1
**Marital status**		
Single	1	0
Married	3	9
Widowed	7	0
Divorced	0	1
**Employment status**		
Unemployed	0	6
Retired	10	4
**Years of caregiving** mean (range)	8 (1–20)	8 (1–20)
**Most common diseases**		
Dementia/ Alzheimer	8	0
Oncologic disease	1	0
Degenerative bone disease	1	6
Depression	0	3
Heart attack and hypertension	0	1
**Barthel Scale - Level of Dependence in self-care**		
High	9	–
Moderate	1	–

Recipients of care ranged from 72 to 93 years, eight were males and two females. Eight had a high level of dependency in self-care according to the Barthel scale: four with 0 score, two with 2 and one 6. Seven had been bedbound for several years. The other two relatives had a moderate level of dependency, scoring 12 and 16 respectively. Only three of the family caregivers were certain that Alzheimer’s was the definitive diagnosis of their relative. The remaining four could not explain what type of dementia their relative had. One patient with the diagnosis of cancer was in end-of-life-care, passing away 3 weeks after family caregiver being enrolled in the study. The rapid progression of the disease led to a sudden process of grief which was not observed in other participants.

### Main findings

Five key overarching themes that were not mutually exclusive were identified by observations, interviews or both: (1) provision of care towards self-care dependence and prevention of complications; (2) perceived needs; (3) unknown needs; (4) caregivers’ own physical and emotional impairments; and (5) balancing limited time. Table 2 shows each theme and associated sub-themes.

**Table 2:** Main themes & subthemes, supported in observations ± interviews

Themes	Sub-themes	Caring activities	Supported in observation	Supported in interviews
Provision of care towards patient self-care and prevention of complications	Family caregivers assistance in activities of daily living	Bathing	✓	✓
		Grooming	✓	✓
		Feeding	✓	✓
		Toileting	✓	✓
		Transfers	✓	✓
		Use of wheelchair	✓	✓
		Socialisation	✓	–
		Help with behaviour management (exercise and diet)	✓	✓
		Help with medical procedures	–	✓
		Promotion in self-care	✓	✓
	Family caregivers assistance towards prevention of complications	Aspiration	–	✓
		Dehydration	–	✓
		Constipation	–	✓
		Moisture lesions	–	✓
		Pressure ulcers	✓	✓
		Ankyloses	–	✓
		Wandering	✓	✓
		Falls	✓	✓
Perceived needs	Knowledge	Prevention of complications	–	✓
		Medicines management	✓	✓
		Management of challenging behaviour	–	✓
		Access to professional support and services	✓	✓
		Understanding process and signs of disease progression and dying	✓	✓
	Practical	Aspiration of secretions	–	✓
		Transfer techniques	✓	✓
		Techniques to prevent pressure ulcers, aspiration and ankyloses	✓	✓
Unknown needs	Prevention of complications	Aspiration, ankyloses, dehydration, constipation and moisture lesions	✓	–
	Knowledge	Disease progression	–	✓
		Signs and symptoms complications	✓	✓
		Medicines and side effects	✓	✓
		Empowerment of patient’s self-care	✓	–
		Access to professional support and services	✓	✓
Family caregivers’ own physical and emotional impairments	Physical	Skeletal muscle health	✓	✓
		Cardiac health	–	✓
	Emotional	Depression	–	✓
		Relationship difficulties	–	✓
Balancing limited time	Caring of other family members	Grand-children, father, brother and husband	–	✓
	Providing income	Raising cattle	–	✓
		Farming	–	✓
	Domestic activities	Preparation of meals	–	✓
		Shopping	–	✓
		Financial management	–	✓
		Washing and ironing clothes	✓	✓
		Cleaning	✓	✓

Legend: subthemes ordered according to the predominance of observations and participant quotes

### Provision of care towards self-care and prevention of complications

Through the observations and interviews the maintenance of self-care of the recipient of care and the prevention of complications was the main and the most noticeable activity provided by family caregivers.

#### Assistance in activities of daily living

Family caregivers were more aware of their role in maintaining self-care than in preventing complications. Data showed that almost all family caregivers assisted the person in activities of daily living ranging from bathing (9/10), grooming (9/10), feeding (8/10), toileting (9/10), medication management (10/10), socialisation (10/10), and help with medical procedures (10/10). The following extracts illustrate this theme.

*She knows how to wash the person, takes care of their skin and hair, and dressing techniques. She is able to adjust the temperature of the water.*

*(Caregiver 1,* Observation field note *3)*

*“I am responsible to lift and transfer her from bed and chair every morning […]. Most of the days, I sit next to her only for her have the feeling of another person.”*

(Caregiver 6, Interview 1)

*“The insulin injection is done by me. I learnt how to do it. Now it is easy.”*

(Caregiver 10, Interview 2)

Transfers of the person to the wheelchair, sofa or bed (9/10) without any human or mechanical assistance were most often observed. During interviews caregivers expressed that they viewed asking another relative or healthcare provider for help as a weakness. Furthermore, most ignored potential aids which might support them in this role.

*“He is too busy [son]. And I don’t need help. I always did it by myself [transfer to bed].”* (Caregiver 2, Interview 1)

Promotion of independence in self-care was a sub-theme, identified in two family caregivers through observations and interviews, although seven relatives could have benefited from this care.

*“And she helps too. [She wets the sponge and I put the soap on [FC]. I reinforce ‘Come on. You do what you can [talking to the relative]’. [FC tries to maintain the physical capacity of the person being cared for*].” (Caregiver 3, Interview 1)

In interviews, when the reason for not stimulating their relative to continue being independent in self-care was discussed, family caregivers gave several reasons: had never thought about that, concerned that the other relatives or neighbours would think that they were neglecting the relative, or it was quicker for family carers to do it themselves than to wait for it to be done by the ill person.

*“I need to feed her. What would other people think if I allow my mom to do this?”* (Caregiver 7, Interview 1)

#### Prevention of complications

Family caregivers were focused on preventing complications including aspiration, dehydration, constipation, pressure ulcers, moisture lesions and wandering. While the encouragement of self-care was evident, interviews around knowledge concerning the prevention of complications were very enlightening. For instance, the researcher needed to understand that the head of the bed was placed at an angle of 30 degrees when the family caregiver put the patient in bed.

*“I position the bed so she doesn't choke ... you know, so the food goes better to the tummy.” (Caregiver 5, Interview 1)*

*PAD was seating in an arm chair, with an immobilizer in place and pillows surrounded her body to avoid a front fall. (Caregiver 1, Observation field note 1)*

A final sub-theme addressed in the observation and interviews was the prevention of falls. All family caregivers were aware of the importance of falls prevention. However, not all had the physical capacity to prevent them. Some were old and had physical impairments of their own. All participants with dementia presented cognitive impairment, which increases the risk of fall. Through observations, only two family caregivers were seen to follow routines and use communication management to avoid further confusion. The only person able to walk (with support of a stick) caused an enormous amount of trouble as she was always trying to escape the home.

*“I spend all day watching her. If I miss a minute, she disappears. One day, I took 2 hours to find her. I do not understand how. She walked almost 2 kilometres with her stick. And she is not able to help me do anything at home. […] I sometimes wish that she was bedbound.”* (Caregiver 9, Interview 1)

Through observations it was evident that once the person cared for became more dependent on self- care the risk of complications increased. Family caregivers were not aware of this complexity, as they continued to support the person in their self-care in the same way they had done until that moment, without noticing the new needs.

#### Perceived needs

Perceived needs were grouped into two sub-themes: knowledge and skills. Both sub-themes were approached in the interviews and informal conversations during the observations, mainly because the researcher felt that these themes were avoided in front of the dependent person. Interviews provided additional insight in relation to knowledge; family caregivers reported the need for a better understanding of the process of dying and disease progression; prevention of complications (pressure ulcers, aspiration etc.); medication and behaviour management; and how to access professional support.

*She knows that the urine becomes more concentrated with age. However, she does not know how much fluid she should give to avoid dehydration. She asks me [researcher] how much fluid she should give and what the signs and symptoms of dehydration are.* (Caregiver 6, Observation field note 2)

Simultaneously, family caregivers talked about the need of skill improvement such as performing suction of oral secretions, how to apply different techniques to transfer the person to wheelchair or toilet seat, and how to provide care to prevent ankyloses, pressure ulcers and aspiration.

*“Do you know that I need to do suction? […] It is not difficult to learn how to use the suction. They explained to my sister first. After that, the nurse came and explained it to me.”* (Caregiver 7, Interview 1)

#### Unknown needs

To identify the unmet family caregivers’ needs, the researcher compared the level of care needed by the person according to palliative care standards to the care provided by the family caregiver. This sub-theme was mainly identified through observations and clarified in interviews. During the study a set of complications that were not prevented was observed. Aspiration, ankylose, dehydration, constipation, moisture lesions, pressure ulcers and falls were not prevented in some instances.

*PAD has high risk of aspiration and the top of bed was less than 30 degrees. However, FC did not do anything. FC did not allow the community nurse to explain how to prevent aspiration. This FC has been caring for more than 10 years and was very upset because the nurse provided an explanation.*

(Caregiver 2, Observation field note 1)

Through the interviews was possible to clarify that these family members ignored these complications or denied they were problematic.

*“I always lay down the bed straightforward breakfast. It is not a problem at all.”*

(Caregiver 2, Interview 1)

Two other sub-themes were identified through observations and interviews. One was the lack of knowledge related to disease progression, warning signs and symptoms, medicines and side effects, exercise and diet and empowerment of self-care of their loved ones. Another sub-theme identified was how carers could access professional support. Five caregivers expressed ignorance of how to obtain health professional support (community nurse, doctors, social workers …), social support (day centres, nursing homes …) and community support. One caregiver called Emergency Medical Services when his wife was dying, because he did not know what was happening.

*“I called to the INEM (ambulance service) that came and took her to the Emergency Medical Services.” (Caregiver 5, Interview 2)*

#### Caregivers’ own physical and emotional impairments

Observations and interviews highlighted other aspects directly related to the family caregivers’ role and capacity to provide care. Some care was not provided due to caregivers’ own physical impairment. For example, support for self-care and prevention of complications were not delivered when the family caregiver experienced physical impairments.

*I asked FC about her tired face. She referred that she has had back pain from an old fracture, and that it took a lot of effort to reposition PAD in bed.*

*(Caregiver 1, Observation field note 4)*

Five of the family caregivers had declining physical capacity were unable to provide physical care. As a consequence, and because they had no access to community or palliative care support, three of the people with advanced disease were entirely bedbound.

*“Before she had been totally bedbound, I bathed her, changed her pad, so on. I was doing everything. But after my heart attack, I started to be weak too and talk to myself ‘No, it can’t be like that. I’m so tired.’. So, I started to care less.*

*(Caregiver 5, Interview 1)*

Two family caregivers shared how their own emotional fragility and lack of relational interaction with their relative interfered with the quality of care provided. Both family caregivers had been followed up by a specialist in psychiatry. Their psychological and emotional state not only interfered with their ability to care but also with their own overall health.

*I went to the psychologist. [...] I went to the psychologist and he told me to stop all the medicine. I just stopped sleeping at night. [...] And then what he advised me to do was to go to a psychiatrist ... First, I still go to the general practitioner (GP) and then I go to the psychiatrist. (Caregiver 9, Interview 1)*

Nevertheless, some of the family caregivers had their own self-care strategies, handcrafting hiking, reading, swimming, going to the hairdresser and watching television. During the observation period some family caregivers gave up their hobbies and free time as the person cared for became more dependent on personal care.

*“I stopped going to the movies, having dinner out. I stopped doing what everyone else does. [...] anywhere I go, I am going to run [trembling voice]. I am experiencing stress for fear of something happening to my mum.”*

*(Caregiver 7, Interview 1)*

#### Balancing limited time

Observations and interviews highlighted lack of time as having a direct impact on the type and quality of care provided. In addition to their caregivers’ roles, these family members were responsible for preparing meals, housekeeping, managing the family budget, raising animals, and cultivating crops for income. Family caregivers expressed that these activities lead to less time to themselves and, consequently, less time available to care for their relative.

*“In addition to taking care of her mother, she supports her daughter by helping to take care of her grandchildren almost every day”*

*(Caregiver 7, Observation field note 2)*

To compensate, they again reduced their leisure activities. Furthermore, in the interviews, other activities were identified that contribute further to the reduction of free time. All caregivers needed to take care of another family member, a father, a husband, grandchildren or a brother. The reason given for this extra care was because they were at home and ´had more free time’.

*"I am responsible for taking care of my father-in-law and my two grandchildren also during the week. I have no time to myself"* (Caregiver 2, Interview 2)

**Fig. 1:**
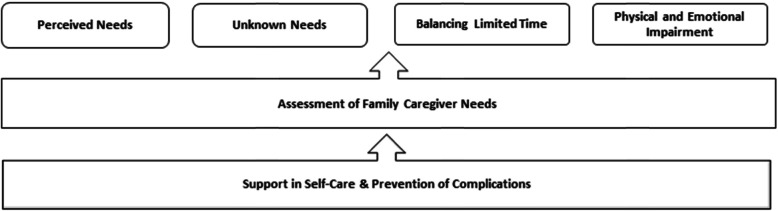
Family Caregivers’ Needs to Support of Relatives with Advanced and Progressive Disease at Home

Figure 1 depicts the main findings, where it is evident that an imbalance in any one of the factors would lead to reduced performance of family caregiver role, and increase the risk of complications for their relatives. This could result in overall decrease in their quality of life for both parties.

## Discussion

This research aimed to explore the experience of family members caring for a relative with advanced progressive disease at home, and identify their care needs. Through the observations, field notes and interviews it was found that most of the care provided is related to maintenance of self-care and prevention of disease complications, which is consistent with previous studies [29]. Despite the challenges, our data reinforce that family caregivers continued caring for relatives who meant losing their free time and physical and emotional health. Providing care at home is a highly demanding task both emotionally and physically [15].

Multiple factors influence the role of family caregivers, what has been reinforce in other international studies: the degree of dependence of the person receiving care, the accessibility of equipment or additional support, the type of relationship between the caregiver and their relative [13, 30, 31]. Furthermore, burn related to lack of time was present and could be minimized if volunteers or relatives could take over same of the tasks performed by family caregiver such as shopping, cooking or laundry. This study shows us how reluctant family caregivers are in accept the support of other family members.

The lack of continuity in health and social care provision appeared to be less coordinated when the caregivers need more support. There could be different reasons why services are not meeting family caregivers’ needs, although the lack of knowledge of how to access service has previously been highlighted in this study as in previous studies [32, 33]. Moreover, nurses should reinforce to family caregivers the importance of asking for support from other relatives or neighbours [34, 35]. For family caregivers who do not have the potential to provide appropriate care or to prevent complications, support might be provided [36].

Improved education, support and understanding of disease progression, medication and symptoms management, warning signs, complications prevention and resources available may help to empower family caregivers’ actions of these participants. The removal of these barriers may support family caregivers to feel more confident in providing care and avoiding complications, hospital admissions as other studies suggest it [15, 34, 37]. Practical training is essential to support family caregivers when seeking help [38]. Besides, family caregivers need advice on how or when to provide care or what devices to use in their caregiving role. Nurses have the opportunity to explain or share information about the awareness of the risk of complications [39]. Healthcare professionals can also deliver training and supervision to increase caregivers’ confidence, prevent complications and burnout [34, 40].

Family nurses’ knowledge and advocacy for policies and programs is also needed to support families, as these programs have a profound influence on them by moderating social determinants of health [41]. Family caregivers’ consolidated knowledge and competences facilitate the interactions and partnerships with professionals to promote better care. This mutual sharing may improve the coordination of care [42]. However, it is also important to clarify the roles and adjust them to caregivers’ individual resources to manage responsibilities in the process of caring.

Although some topics have been found in both observations and interviews, there were significant differences. Interviews allowed us to identify the perceived needs, which had not been possible with most observations. Furthermore, interviews reinforced some observations. For example, it was evident how lack of time can interfere with the quality of life of the family caregiver [43]. Moreover, family caregivers sometimes expressed their difficulties in managing their own emotions and feelings during the interview, which was difficult to identify through observations.

However, initially, unmet needs and lack of knowledge were only identified by the observations and later clarified through interviews. This lack of awareness might explain why some family caregivers became irritable and hostile and blamed others for the difficulties they had been experiencing [44]. These behaviours can be explained by their perceived stress. A comprehensive, tailored psychosocial intervention with supervision, support and consultation would be crucial to family caregivers but should be adapted to cultural environment [45]. If psychoeducational and emotional support is required, community nurses are the best placed healthcare professionals to provide this kind of support [4]. Advice from professionals, counselling, small videos with demonstrations or educational modules can be provided on this basis and can reduce family caregivers’ stress and anxiety [46]. Psychoeducational support is an important factor in the promotion of safety practices, skills and knowledge development. By empowering persons who use health and social care services through research, a course can be provided to influence change and improve the issues which concern people the most [47].

These findings have implications for clinical practice, policy and research. Nurses have an opportunity here to use the best evidence in their daily practice and increase the knowledge of their discipline [48]. Health care professionals have the duty to provide families with the best care, supplying, at the same time, an essential basis for informing research programs and individual studies [49]. In this study, only some of the participant had a pro-active support from their community nurse and most related to the care provided than to obtain personal support to their own needs. Healthcare professionals should consider family caregivers, not just as a support for the ill person but as providers rendering a service in their own right. Moreover, clinical documentation should support nurses in their decision-making throughout the evaluation process. Nurses ought to spend and value time with family caregivers, observing and conversing with them to gain an in-depth understanding of their needs – as family caregiver and relative. The information obtained may support the decisions to provide the best care aimed to improve their quality of life.

An understanding of the family caregiver needs over time, particularly outside of high-income settings, ought to improve care delivery and quality of patient and family caregiver care. Our findings emphasise family caregivers should be seen as equal partners. Furthermore, family-focused care would enhance nursing practice in this context and this research can inform nursing training and educational programs.

Further research should focus on the developing tools to help caregivers cope and manage their own needs and those of their beneficiaries. These tools need to “fit” into individual needs and be integrated into everyday activities. They should be culturally-sensitive and family-oriented to improve the quality of life of family caregivers and their families, moving towards a palliative care approach, family focused, and take a holistic, person centred perspective. These instruments ought to release health professionals to undertake other tasks. Evidence from other studies demonstrates that the provision of new resources, such as health technology, support health professionals and the interaction between health professionals and family caregivers [50]. The use of educational technologies could also complement the support offered by healthcare professionals.

### Limitations

Despite valuable findings of this study, there are several limitations that need to be recognised. The findings only represent the perspectives of family caregivers and not the relatives’ own perspective. It should be considered that all the data emerged from the same context of care. Furthermore, a more detailed contextual information could have been obtained, for example, income sources determinants; health and social care professional support; and financial burden. These findings only reflect participants from a rural area who lacked palliative care support. They may not be generalised or reflect the care provided by family caregivers elsewhere in Portugal or internationally. However, our findings are supported by previous studies as cited above, reinforcing their reliability and transferability.

## Conclusions

This research provides a detailed understanding of family caregivers’ needs when caring for a relative with advanced disease at home. Family caregivers of people with advanced progressive illness are required to balance provision of care towards independence and prevention of complications with their own needs and impairments with limited time. The findings underscore the importance of family-focused care and the adoption of a family perspective. Families should be seen as equal partners in the process of decision making.

### Recommendations

Healthcare professionals must strive for a comprehensive assessment of the caregiver-patient dyad and collaborative working with the caregiver who can embrace their role. This process requires time and potentially repeated visits but can provide key information to inform personalised care plans and improve the quality of care provided. This approach could be a means to change the way in which health and social care services deliver holistic and collaborative care. Widespread adoption of a family perspective would enhance nursing practice in this context. Comprehensive assessment of family caregiver needs should be included in nursing education and training programs.

## Supplementary information

**Additional file 1: Table S1.** Report in accordance with the COREQ guidelines – checklist for reporting qualitative research.

## Data Availability

The data set used and/ or analysed during the current study are available from the corresponding author on reasonable request.

## Abbreviations

COREQ: COnsolidated criteria for Reporting Qualitative research
FC: Family caregiver
GP: General Practitioner
INEM: Instituto National de Emergência Médica
PAD: Person with Advanced Disease
