# Supplementary material for: Understanding family caregivers’ needs to support relatives with advanced progressive disease at home: an ethnographic study in rural Portugal
Source: BMC Palliat Care. 2020 May 25;19:73. doi: 10.1186/s12904-020-00583-4 (PMC7249372; doi:10.1186/s12904-020-00583-4)
Supplement: Supplementary file 1 — Additional file 1: Table S1. Report in accordance with the COREQ guidelines – checklist for reporting qualitative research. [file 12904_2020_583_MOESM1_ESM.docx]

**Table** **S1** Report in accordance with the COREQ guidelines – checklist for reporting qualitative research

| **No item** | **Description** |
| --- | --- |
| ***Domain I: research team and reflexivity*** |  |
| **Personal characteristics** |  |
| 1. Interviewer/ facilitator | M.J.T. conducted all interviews and observations. |
| 2. Credentials | The first author is a nurse and got a PhD in Education and a Master in Bioethics. She is a clinical nurse specialist in Medical Surgery; W.A. and N.C. hold both a PhD and work as full professors at the Porto School of Nursing and University of Aveiro, Portugal, respectively. M.M. is a Senior Lecturer in Health Services Research and Specialist Physiotherapist in Palliative and End of Life Care. |
| 3. Occupation | M.J.T. worked with Health Computer Systems in the Hospital São João – Porto, Portugal and currently works as a research nurse, and M.M as a Senior Lecturer at the Cicely Saunders Institute, King’s College London, United Kingdom. |
| 4. Gender | M.J.T. and N. C. are female and W. A. and M.M. are male. |
| 5. Experience and training | The main investigator M.J.T. is an expert in International Classification for Nursing Practice, advanced nursing and medical surgery and has experience in research in different areas including quantitative and qualitative research. She concluded a Master degree (MSc) in the Catholic University of Portugal in 2006. In addition, she underwent formal PhD Education in qualitative research with specialization in supervision and assessment in 2016. W.A. and N.C. are University Full Professors and have experience on advising research related to family caregivers of dependent people at home. W.A. is an expert on palliative care and dementia issues. Currently, he is working on Best Practice Statements for people with advanced dementia. M.M is an expert in function and rehabilitation in palliative care. |
| **Relationship with participants** |  |
| 6. Relationship established | There was no relationship between the research / interviewer and participants. No participants were recruited from the hospital where M.J.T. was working as a nurse to avoid ethical problems and bias grounded on dependence issues. |
| 7. Participant’s knowledge of the interviewer | Participants were informed that the interviewer was a researcher in the University of Aveiro and that the goal of the research was the investigation of family caregivers of person cared at home, including observation of their tasks, their opinion about perceived needs, support and unmet needs according to the condition of people receiving care. When the family caregivers asked, M.J.T. explained more about her background as a researcher and a nurse. |
| 8. Interviewer characteristics | The main interest of M.J.T. in the topic was grounded in her daily work and experience in a hospital where the challenges related to family caregivers and end-of-life care after discharge emerges every day. |
| ***Domain 2: study design***  **Theoretical framework** |  |
| 9. Methodological | A qualitative approach guided by the Spradley participant observation perspective. The first part of this study focused on exploring what kind of care was provided by family caregivers, what have been their perceived needs and what elements should be included into psychoeducational programs. In-depth observations and interviews were undertaken between December 2012 and August 2013, with family caregivers of people who were receiving care at home. |
| **Participants selection** |  |
| 10. Sampling | Convenience sampling on a rural area was used in this study. All approached family caregivers agreed to participate. |
| 11. Method of approach | The Unit indicated two key informants who gave the basic information about the participants, prepared the visits on their homes and facilitated the interactions. Moreover, the key informants were crucial to explain the caregivers what role the research would play in the field, to ensure the confidentiality of the collected information and to discuss with the researcher the study findings. |
| 12. Sample size | In total 10 family caregivers participated in the study. A convenience sample was used. |
| 13. Non-participation | None of the participants refused or withdrew from the study. 2 participant passed away during the study. |
| **Setting** |  |
| 14. Setting of data collection | Data were collected in the homes of people receiving palliative and end-of-life care. All interviews were conducted in private without participation of staff, patients or other family members. All the observations were conducted also at home of the person in palliative care pathway, with the consent of family caregiver. Data collection was terminated due to data saturation. |
| 15. Presence of non-participants | No one else was present besides the participants and the researcher. |
| 16. Description of sample | Our sample is described in the ‘Methods’ section. Table 2 shows participant characteristics. |
| **Data collection** |  |
| 17. Interview guide | Semi-structured interviews were conducted in the second phase and schedule was developed using a literature review. Table 1 shows the topics included in the interview guide. |
| 18. Repeat interviews | Only one interview was repeated because the participant suggested continuing in another day. |
| 19. Audio/visual recordings | All interviews were digitally recorded and stored on a computer according to the rules, regulations, and recommendations of the regional ethics committee. |
| 20. Field notes | Researcher M.J.T. made field notes during the interviews and observations. These include personal impressions and other observations that were not recorded. Field notes were used in the analysis of the questions and prove the findings. |
| 21. Duration | The duration of interviews and observations varied from 30 to 60 minutes. |
| 22. Data Saturation | Data saturation was reached from family caregivers’ interviews and observations. |
| 23. Transcripts returned | Due to practical reasons (functional illiteracy of some participants), transcripts were not returned to them to comment. |
| ***Domain 3: analysis and findings*** |  |
| 24. Data analysis | M.J.T. performed data codification. W.A. and N.C. validated the process. |
| 25. Description of the coding tree | Themes derived from data. A coding tree was defined. |
| 26. Derivation of themes | Themes emerged from data and were discussed and agreed on by M.J.T. and W.A. |
| 27. Software | Verbatim transcription of the digital interview recordings was done by M.J.T. Analysis and coding of the transcripts was supported by the software WebQDA (developed by the University of Aveiro). |
| 28. Participants checking | There was no feedback from the participants on the findings (due to practical reasons as explained above). At the end of the interviews and observations, the researcher gave a short summary of the interview/ observations content and asked for clarifying questions. This enabled the informant to check whether the researcher had understood the main content right. |
| **Reporting** |  |
| 29. Quotations presented | Themes are presented and illustrated by participant quotations that are identified by a participant number as well an observation or interview number. Participant number does not correspond to the number displayed in Table 2 to protect participants and ensure confidentiality. |
| 30. Data and findings consistent | Data and findings herewith are consistent with our point of view, especially when we used the triangulation of methods to collect them. |
| 31. Clarity of major themes | The major themes are presented in the results/ findings and illustrated in table 3. |
| 32. Clarity of minor themes | Minor themes are described in the result chapter. |
